# Effects of pre-emptive pregabalin and multimodal anesthesia on postoperative opioid requirements in patients undergoing robot-assisted laparoscopic prostatectomy

**DOI:** 10.1186/s12894-021-00785-9

**Published:** 2021-02-02

**Authors:** K. Sisa, S. Huoponen, O. Ettala, H. Antila, T. I. Saari, P. Uusalo

**Affiliations:** 1 Perioperative Services, Intensive Care and Pain Medicine, Turku University Hospital, Turku, Finland; 2 Department of Anaesthesiology and Intensive Care, University of Turku, Kiinamyllynkatu 4-8, P.O. Box 51, 20521 Turku, Finland; 3 Department of Urology, University of Turku, Turku, Finland

**Keywords:** Robot-assisted laparoscopic prostatectomy, Multimodal analgesia, Anesthesia

## Abstract

**Background:**

Previous findings indicate that pre-emptive pregabalin as part of multimodal anesthesia reduces opioid requirements compared to conventional anesthesia in patients undergoing robot-assisted laparoscopic prostatectomy (RALP). However, recent studies show contradictory evidence suggesting that pregabalin does not reduce postoperative pain or opioid consumption after surgeries. We conducted a register-based analysis on RALP patients treated over a 5-year period to evaluate postoperative opioid consumption between two multimodal anesthesia protocols.

**Methods:**

We retrospectively evaluated patients undergoing RALP between years 2015 and 2019. Patients with American Society of Anesthesiologists status 1–3, age between 30 and 80 years and treated with standard multimodal anesthesia were included in the study. Pregabalin (PG) group received 150 mg of oral pregabalin as premedication before anesthesia induction, while the control (CTRL) group was treated conventionally. Postoperative opioid requirements were calculated as intravenous morphine equivalent doses for both groups. The impact of pregabalin on postoperative nausea and vomiting (PONV), and length of stay (LOS) was evaluated.

**Results:**

We included 245 patients in the PG group and 103 in the CTRL group. Median (IQR) opioid consumption over 24 postoperative hours was 15 (8–24) and 17 (8–25) mg in PG and CTRL groups (*p* = 0.44). We found no difference in postoperative opioid requirement between the two groups in post anesthesia care unit, or within 12 h postoperatively (*p* = 0.16; *p* = 0.09). The length of post anesthesia care unit stay was same in each group and there was no difference in PONV Similarly, median postoperative LOS was 31 h in both groups.

**Conclusion:**

Patients undergoing RALP and receiving multimodal analgesia do not need significant amount of opioids postoperatively and can be discharged soon after the procedure. Pre-emptive administration of oral pregabalin does not reduce postoperative opioid consumption, PONV or LOS in these patients.

## Background

Prostate cancer is the second most frequent malignancy among males worldwide [1]. Robotic-assisted laparoscopic radical prostatectomy (RALP) has evolved into the predominant surgical approach in localized prostate cancer. Due to its minimally invasive nature, RALP is associated with decreased pain levels compared to open prostatectomy (ORP) [2]. Despite its minimally invasiveness, patients undergoing RALP experience mild to moderate postoperative pain and often need opioids perioperatively [3, 4].

Standardized, multimodal analgesic regimen with non-opioid agents aims to minimize the use of opioids and thereby decrease opioid-related adverse effects [5]. A recent review concluded that multimodal analgesia is readily available and the evidence to support its efficiency is strong [6]. However, the effect of current analgesia regimen is poorly studied and to our knowledge, there is only one study evaluating the multimodal analgesic regimen in patients undergoing RALP [7]. A recent systematic review on the perioperative pain regimen for radical prostatectomy surgery concluded a need to develop an optimal pain management protocol in this patient population [8].

Pregabalin is a gamma-aminobutyric acid analogue that binds to voltage-gated calcium channels and decreases pain sensation by inhibiting calcium influx and the subsequent release of excitatory neurotransmitters in the central nervous system. Previously, multiple systematic reviews and meta-analyses have concluded pregabalin to cause an opioid sparing effect during the perioperative period in several patient groups [9–11]. Pregabalin has been used for as premedication in patients undergoing RALP [7]. However, recent studies have brought up concerns about respiratory depression in patients that received pregabalin in combination with opioids and/or general anesthetics [12, 13]. While pregabalin predisposes analgesic effects, the use of pregabalin as a routine component of premedication has been challenged due to the imbalance of clinical advantages and possible side effects [14]. Thus, re-evaluation of the effect of pregabalin on RALP is justified.

The primary objective of our study was to evaluate the impact of single dose of pre-emptive pregabalin on postoperative opioid consumption of RALP patients receiving multimodal analgesia. We hypothesized that the cumulative consumption of opioids after RALP is modest and pregabalin would cause no meaningful effect on postoperative opioid consumption. Our secondary aim was also to evaluate the efficacy of our perioperative multimodal analgesic regimen of RALP.

## Methods

### Patient population

Eligible patients were identified from the RALP register of Department of Urology, Turku University Hospital. Patient data were retrieved from patient database and anesthesia reports of the hospital. Patients scheduled for RALP under multimodal anesthesia in Turku University Hospital (tertiary hospital), South-West Finland between 2015 and 2019 were retrospectively screened and patients with ASA status 1–3, age between 35 and 80 years, weight between 50 and 100 kg were included in the study.

Exclusion criteria included: patients receiving other adjuvant analgesics such as clonidine or tricyclic antidepressants pre-, intra- or postoperatively, patients with chronic opioid or gabapentinoid use, patients with history of chronic pain syndrome, perioperative abnormalities or patients with kidney failure (creatinine clearance < 90 ml/min), patients with abnormal liver function tests or patients with clinically significant abnormalities in preoperative medical examination, ECG or laboratory values and patients undergoing total intravenous anesthesia.

Consecutive patients who met the inclusion criteria and received 150 mg of pregabalin for premedication were identified between January 2015 and December 2017 (pregabalin group; PG). Based on controversial reports regarding use of pregabalin [13, 14] this premedication was discontinued in the beginning of 2018 and consecutive patients who met the inclusion criteria and did not receive any pregabalin during their treatment were identified between between January 2018 and August 2019 (control group; CTRL).

### ERAS protocol

Patients were allowed to take clear liquids until 2 h before the procedure. Multimodal anesthesia with restricted fluid protocol (app. 5 ml/kg/h) was used. Nasogastric tubes were used intraoperatively and removed before the extubation. Perioperative pain was managed with several non-opioid agents (see “Anesthesia management” and “Management of postoperative pain and nausea” sections) Opioids were given only on demand only for stronger pain. Patients were allowed to take normal diet starting from 4 h after discharge from the PACU and encouraged to walk in the evening of the operation day.

### Surgical technique

RALPs were performed by four experienced urologists all performing at least 50 prostatectomies a year. The procedure were done per routine using a technique originally introduced by Abbou et al. [15]. If feasible, bladder neck was preserved and/ or a uni- or bilateral nerve sparing was performed. Also, extended lymphadenectomy was performed in selected cases [16]. For reconstruction, a Rocco stich was used [17]. Also, in the end of the surgery, all patients received an infiltration anesthesia with 40 ml of 0.75% Ropivacaine to troacar openings. Blood loss was measured intraoperatively by taking account the amount of blood in suction and the weight of the cloths.

### Anesthetic management

All patients received preoperatively 1500 to 2000 mg of paracetamol orally and 90 to 120 mg of etoricoxib orally according to their weight. Higher dose for patients over 90 kg and lower dose for patients less than 90 kg. PG group received additional 150 mg of pregabalin orally one hour before the anesthesia induction. The fixed dose of pregabalin was based on previous study on pre-emptive multimodal analgesia of RALP [7]. General anesthesia was induced with intravenous propofol and intravenous fentanyl and maintained with volatile anesthetics (sevoflurane or desflurane). Patients received intraoperatively 8 mg of intravenous betamethasone, 25 mg of intravenous esketamine, 0.75 mg of intravenous dehydrobenzperidol and 2500/10 mg of intravenous metamizole-pitofenone combination (Litalgin®). Neuromuscular blockade was induced with rocurone and reversed with neostigmine-glycopyrrollate. We monitored the depth of anesthesia with entropy (GE B850 Monitor Entropy Module, Helsinki, Finland) and our aim was to keep the target state entropy (SE) between 35 and 45. Intraoperative mean arterial pressure (MAP) target was between 65 and 75 mmHg depending on the patients age and disease history.

### Management of postoperative pain and nausea

In postoperative anesthesia care unit (PACU) a standard pain therapy regimen based on intravenous oxycodone was used. Oxycodone was administered at the dose of 0.03–0.05 mg/kg if patient reported moderate or intense pain (Visual Analog Scale; VAS > 3). Postoperative nausea and vomiting (PONV) was treated with 4 mg of intravenous ondansetron. If patients received antiemetics postoperatively, it was considered as PONV, and if patients received repeated doses of ondansetron or other antiemetics, it was considered as severe PONV.

In the ward all patients received 1000 mg of paracetamol three times a day for postoperative pain. More intense pain (VAS > 3) was managed with 0.05–0.1 mg/kg of oral oxycodone.

### Time to discharge

Time to the discharge was defined as the period of time between the end of surgery and the time of discharge of the patient from the urologic inpatient ward. Clock times were obtained from the hospital’s patient information system.

### Statistics

The primary outcome variable was the cumulative amount of opioids administered to the patients (mean equivalent dose; MED) in PACU and within 12 and 24 h after the end of surgery [17]. A 25% reduction in opioid use was considered clinically feasible. For a study power of 80% (α = 0.05, β = 0.2), the required sample size per group was calculated to be at least 75. Secondary outcomes were the incidence of PONV and severe PONV, PACU time, LOS, duration of surgery (including docking time), intraoperative fentanyl consumption and intraoperative blood loss. The Shapiro–Wilks test (*p* > 0.05) and Q–Q-plots were used to assess normality assumptions. Student’s *t* test was used to compare the groups with normally distributed data, and Wilcoxon’s rank sum test was used to test non-normally distributed data. Nominal data were tested using chi-square analysis. *P* < 0.05 (two-tailed) was considered statistically significant. The results are expressed as mean values with standard deviations (SD), and as medians with interquartile ranges (IQR) when the normality assumption was not met. The covariate effect on postoperative opioid consumption was modelled using linear mixed model for repeated measurements. Time effect was handled as continuous to compare the slope of cumulative opioid dose between the groups (group * time interaction) adjusted with age, BMI, intraoperative fentanyl requirement, operative time as continuous covariate; pelvic lymph node dissection and group as categorical explanatory variables. Compound symmetry was selected for covariance-variance structure in the model. To fulfill assumption for normality a square root transformation was used and the normal distribution assumption was checked using studentized residuals. The analyses were performed with JMP Pro 13.0 and SAS® System programs, version 9.4 for Windows (SAS Institute Inc., Cary, NC, USA).

## Results

245 consecutive patients were included in the PG group and 103 consecutive in the CTRL group (Additional file 1: Fig. S1). There was no difference in the patient characteristics (Table 1).

**Table 1:** Patient characteristics

	PG-group (n = 245)	CTRL-group (n = 103)	*p* value
Age (y)^a^	66 (61–69)	66 (61–69)	0.96
Weight (kg)^b^	85 (12)	84 (11)	0.79
BMI (kg/m^2^)^b^	26 (3)	26 (3)	0.83
Gleason			0.57
6 (n (%))	49 (20)	17 (17)	
7–8 (n (%))	165 (67)	75 (73)	
9–10 (n (%))	31 (13)	11 (11)	
T-class			0.41
T1 (n (%))	91 (37)	37 (36)	
T2 (n (%))	103 (42)	48 (47)	
T3 (n (%))	49 (20)	17 (17)	
T4 (n (%))	2 (1)	1 (1)	
Duration of surgery (min)^a^	163 (143–195)	169 (146–198)	0.22
Intraoperative blood loss (ml)^a^	81 (50–150)	100 (50–150)	0.497
Lymph node extraction (n (%))	108 (44.1)	45 (43.7)	0.94

^a^Median and interquartile range (IQR)

^b^Mean and SD

**Fig. 1:**
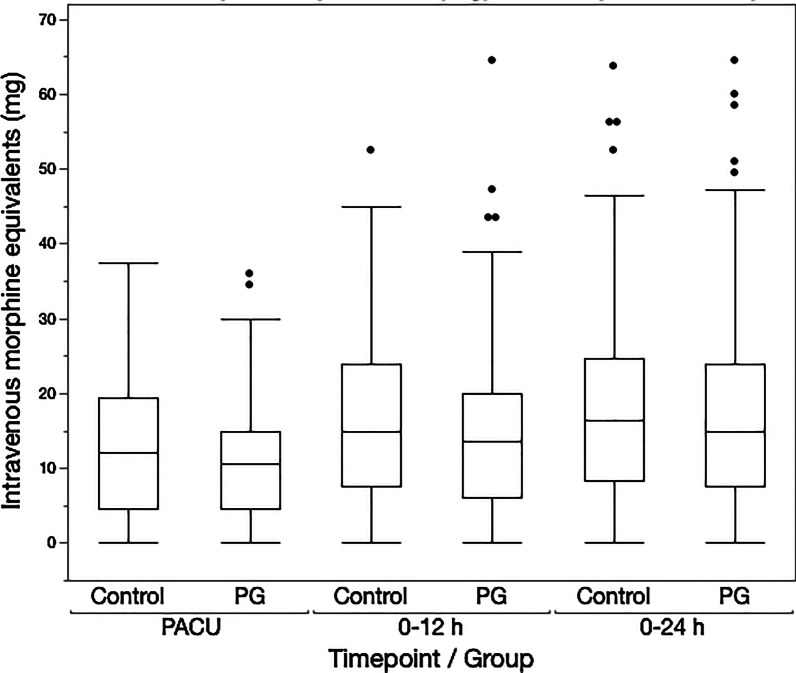
Postoperative opioid consumption in oral morphine equivalents (mg) within three postoperative time periods

There was no difference in postoperative opioid consumption between the groups. Median (IQR) opioid consumption was in 11 (5–20) versus 12 (5–20) mg in PACU (median difference 1.5 mg, 95% CI − 3.0 to 4.5 mg, *p* = 0.16), 14 (6–21) versus 15 (8–24) within 12 postoperative hours (median difference 1.5 mg, 95% CI − 1.0 to 6.0 mg, *p* = 0.09), and 15 (8–24) versus 17 (8–25) mg within 24 postoperative hours in PG and CTRL groups respectively (median difference 1.5 mg, 95% CI − 1.0 to 7.5 mg, *p* = 0.44) (Fig. 1).

There was no difference in PONV (*p* = 0.34), severe PONV (p = 0.84), PACU stay (*p* = 0.18), LOS (*p* = 0.62), time of anesthesia (*p* = 0.22), intraoperative fentanyl consumption (*p* = 0.06), intraoperative blood loss (*p* = 0.497) or in opioid requirement on first postoperative day between the two groups (Table 2).

**Table 2:** Outcomes of the study

	PG-group (n = 245)	CTRL-group (n = 103)	*p* value
Intraoperative fentanyl requirement (mg)^a^	400 (350–450)	350 (300–400)	0.06
Postoperative opioid consumption in PACU (mg)^a^	11 (5–20)	12 (5–20)	0.16
Postoperative opioid consumption 0–12 h (mg)^a^	14 (6–21)	15 (8–24)	0.09
Postoperative opioid consumption 0–24 h (mg)^a^	15 (8–24)	17 (8–25)	0.44
PACU time (min)^a^	128 (108–152)	126 (103–145)	0.18
LOS (h)^a^	31 (28–49)	31 (28–48)	0.62
PONV (n (%))	19 (7.6)	7 (6.6)	0.34
Severe PONV (n (%))	4 (1.6)	2 (1.9)	0.84
Opioid requirement on first postoperative day (n (%))^b^	79 (32.2)	37 (35.9)	0.36

^a^Median and interquartile range (IQR)

^b^mean and SD

In a subgroup analysis of patients undergoing lymph node extraction, there was no difference in the opioid requirement 0–48 h postoperatively in the CTRL group compared with the CTRL group (*p* = 0.94). Similarly, in a subgroup analysis of patients not undergoing lymph node extraction, there was no difference in the opioid requirement 0–24 h postoperatively in the PG group compared with the CTRL group (*p* = 0.34).

In a multivariate analysis intraoperative time was associated with postoperative opioid consumption and age was negatively associated with postoperative opioid consumption. There was no association between postoperative opioid consumption and BMI, intraoperative fentanyl consumption or lymph node extraction. There was no difference in the postoperative opioid consumption between the groups (Table 3).

**Table 3:** Summary of final repeated-measures mixed models of the effect of group

	Postoperative opioid consumption		
Independent variable	DF	F value	*p* value
Age	339	28.03	< 0.001
Group	383	2.51	0.11
Timepoint	690	289.11	< 0.001
Timepoint*Group	690	0.88	0.35
BMI	339	1.24	0.27
Intraoperative fentanyl	339	0.72	0.4
Intraoperative time	339	4.20	0.04
Lymph node extraction	339	1.12	0.29

Data show the effect of group on postoperative opioid consumption taking into account age, BMI, intraoperative fentanyl consumption, intraoperative time and lymph node extraction. Models were run using linear mixed model for repeated measurements

### Adverse events

There were no adverse events recorded.

## Discussion

Our results indicate that pre-emptive administration of oral pregabalin does not reduce postoperative opioid consumption, PONV, PACU time or LOS in patients undergoing RALP. Considering the possible side effects related to use of pregabalin [14], our results do not encourage to use pregabalin in patients undergoing RALP. Instead we were able to demonstrate the feasibility of a new multimodal analgesic protocol that has not been earlier introduced for patients undergoing RALP. Patients receiving this regimen needed only small amount of opioids for postoperative pain and can be discharged soon after the procedure.

The groundwork of multimodal analgesic regimen is formed by paracetamol in combination with a non-steroidal anti-inflammatory drug (NSAID) or a cyclooxygenase 2 (COX2) inhibitors, when no contraindications are encountered, and local anesthesia—a combination proposed by the guidelines from the American Society of Anesthesiologists task force on acute pain management [18] Ketamine, a traditional anesthetic, has been shown to be an effective adjunct for postoperative analgesia at low doses [19]. Systemic corticosteroids, like dexamethasone are often used for the prevention of PONV, but have also been shown to reduce postoperative pain in many types of surgery [20].

Trabulsi et al. [7] demonstrated in their retrospective study a positive effect of multimodal analgesia on postoperative opioid consumption after RALP. In addition to paracetamol and celecoxib (COX2-inhibitor), their regimen included 150 mg single-dose of pregabalin as premedication. Compared to conventional analgesic regimen use of above-mentioned premedication decreased postoperative opioid consumption. In more recent meta-analyses pregabalin as a component of postoperative acute pain management protocol has been generally questioned. While pregabalin may have a minimal opioid-sparing effect, the risk of serious adverse effects seems to be increased and a routine use of pregabalin for postoperative pain treatment cannot be recommended [14, 21].

Our multimodal analgesia regimen included esketamine, betamethasone and metamizole-pitofenone. Pre-emptive use of esketamine for postoperative pain has been studied in several patient groups and there is strong evidence suggesting its use intraoperatively [22]. Use of glucocorticoids as an adjunct to general anesthesia has been shown to decrease postoperative pain, but glucocorticoids have previously studied mainly in orthopedic patients [23]. There is also evidence that intraoperative use of betamethasone decreases PONV after general anesthesia [24]. Single dose metamizole has been shown to have good analgesic effects on postoperative pain [25] and it is often administered together with a spasmolytic compound pitofenone. The above mentioned intraoperative multimodal analgesia has been used in Turku University Hospital for RALP patients over 8 years.

Multimodal analgesia often includes also utilization of local anesthetic-based regional analgesic techniques. In our multimodal analgesia protocol patients received local anesthetic infiltration to the troacar openings. Transversus abdominis plane block (TAP) has been recently introduced as part of multimodal anesthesia and analgesia of RALP [26]. TAP has been shown to reduce pain and opioid consumption after RALP [27] and may be done by the surgeon under visual control or by anesthesiologist under ultrasound guidance. However, to reach a high success rate, this procedure requires training [28].

Due to its minimally invasive nature, the RALP is associated with decreased pain levels compared to open prostatectomy [3]. Immediately after RALP, the main source of pain/discomfort is abdominal, followed by catheter related, penile and bladder-spasm-related discomfort. With current analgesic regimens, abdominal pain after RALP is mild to moderate—on average rated 3 to 4 of 10 on a pain scale [4]. A recent systematic review on the optimal perioperative pain regimen for radical prostatectomy concluded that there is a lack of evidence to develop an optimal pain management protocol in this patient population and specific studies comparing pain and analgesic requirements for open and minimally invasive surgical procedures are warranted [8]. In the face of current opioid crisis, an attempt to minimize the use of opioids perioperatively should become a part of standard care for all surgical patients [29, 30].

The majority of patients undergoing RALP at high-volume centers are discharged on the first postoperative day [31]. In our study the median of LOS was 31 h, which is in line with earlier findings. Postoperative pain was well controlled and major part of patients did require opioids on first postoperative day. The main reasons for discharge later than on the first postoperative day were logistical i.e. patient living in the rural areas needing a special means of transportation or suspicion of acute postoperative complication such as bleeding or infection.

Perioperative dosing of pregabalin has been recently surveyed in various laparoscopic surgery patient populations. In a very recent randomized controlled trial patients undergoing laparoscopic colorectal surgery and receiving two doses of oral pregabalin had lower postoperative opioid consumption but similar pain scores compared to control group [32]. A prospective study with patients undergoing laparoscopic living donor nephrectomy receiving two doses of oral pregabalin had lower postoperative opioid consumption but similar pain scores compared to the control group [33]. Contrary to these, a recent randomized controlled trial with similar setting to our study concluded that pregabalin together with celecoxib offered no analgesic superiority over standard opioid care in postoperative pain therapy following laparoscopic cholecystectomy. Recent large systematic review including 39 trials on endoscopic abdominal surgery found no clinically significant analgesic effect for perioperative used gabapentinoids. Use of perioperative pregabalin was associated with greater risk of adverse events [34]. Our findings together with this systematic review suggest that routine use of pregabalin for patients undergoing laparoscopic surgery cannot be recommended.

Our retrospective study has some limitations. First, while patients after RALP experience mild to modest pain and the amounts of opioids are modest as well, it is difficult to demonstrate meaningful difference. Furthermore, owing to the lack of data, we were not able to reliably assess the postoperative pain scores, or the amount of opioid use after discharge. For the same reason, we were not able to assess the incidence of chronic pain and hyperesthesia in the long term. The retrospective design of the study could have also affected the results, even when only consecutive patients were included to avoid any selection bias. On the other hand all patients in our study received a standardized multimodal anesthesia and only few experienced surgeons were involved in the procedure. Moreover, we were able to demonstrate an effective multimodal anesthesia protocol associated with few side effects and a short LOS.

To strengthen the findings of our study, the effect of pregabalin on postoperative opioid consumption and pain of patients undergoing RALP could be studied in a prospective manner. Another medication worth giving an opportunity as a part of multimodal analgesic regimen would be alpha-2-agonist clonidine, which has been shown to reduce postoperative opioid consumption [35, 36], but has not been studied in patients undergoing RALP. While deep Trendelenburg position during RALP often comes with a rise in mean arterial blood pressure [37], clonidine’s ability to provide perioperative hemodynamic stability could be of use.

According to our findings, patients undergoing RALP that receive paracetamol and etoricoxib as premedication, esketamine, betamethasone, metamizole-pitofenone, fentanyl and local infiltration anesthesia intraoperatively, and paracetamol for postoperative pain need only small amounts of opioids postoperatively. While our findings demonstrated that routine use of pre-emptive pregabalin does not decrease postoperative opioid consumption in patients undergoing RALP, perioperative use of pregabalin may still have potential benefits in patients with a history of neuropathic or chronic pain.

## Conclusion

Pre-emptive administration of oral pregabalin does not reduce postoperative opioid consumption, PONV, PACU time or LOS in patients undergoing RALP. Our findings indicate that patients receiving multimodal analgesia can be discharged soon after the procedure and require less opioids for postoperative pain.

## Supplementary Information

**Additional file 1: Figure 1.** Flow diagram of the study.

## Data Availability

The data that support the findings of this study are available from the datasets of the Department of Urology and Department of Anesthesiology and Intensive Care and the Informatics Department of Turku University Hospital, but restrictions apply to the availability of these data, which were used under license for the current study and are not publicly available. However, data are available from the authors upon reasonable request and after permission of the Ethics Committee from Southwest Finland Hospital District.

## Abbreviations

RALP: Robot-assisted laparoscopic prostatectomy
PG: Pregabalin group
CTRL: Control group
PONV: Postoperative nausea and vomiting
LOS: Length of stay
ORP: Open prostatectomy
PACU: Post anesthesia care unit
NSAID: Non-steroidal anti-inflammatory drug
COX2: Cyclooxygenase 2 inhibitor
VAS: Visual analog scale
MED: Mean equivalent dose of morphine
