# Supplementary figures and images for: Effects of pre-emptive pregabalin and multimodal anesthesia on postoperative opioid requirements in patients undergoing robot-assisted laparoscopic prostatectomy
Source: BMC Urol. 2021 Feb 2;21:14. doi: 10.1186/s12894-021-00785-9 (PMC7856812; doi:10.1186/s12894-021-00785-9)

## Flow Diagram

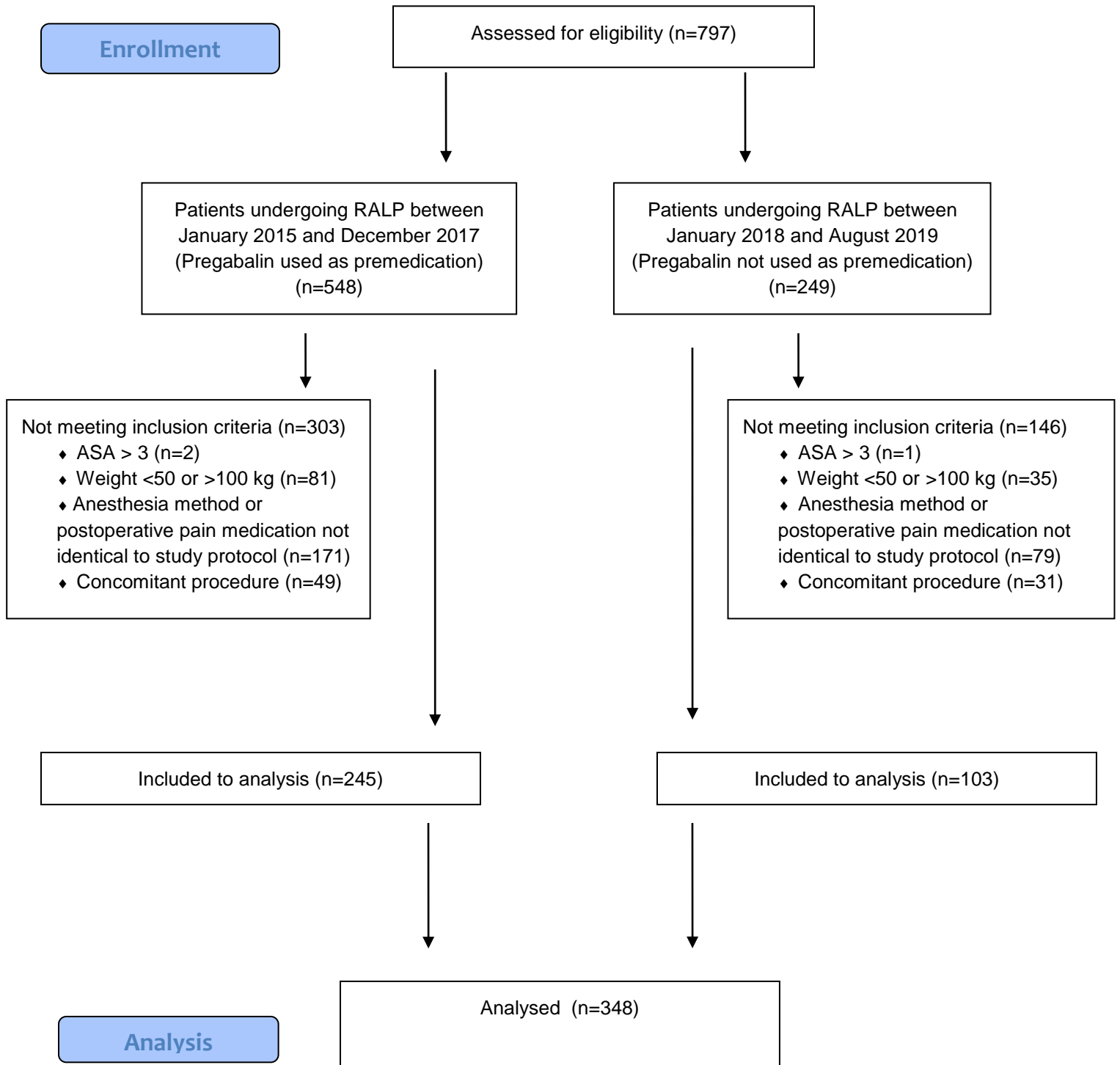

Supplement: Supplementary file 1 — Additional file 1: Figure 1. Flow diagram of the study. [file 12894_2021_785_MOESM1_ESM.pdf]
